# Review of the Quality Control Checks Performed by Current Genome-Wide and Targeted-Genome Association Studies on Myalgic Encephalomyelitis/Chronic Fatigue Syndrome

**DOI:** 10.3389/fped.2020.00293

**Published:** 2020-06-12

**Authors:** Anna D. Grabowska, Eliana M. Lacerda, Luís Nacul, Nuno Sepúlveda

**Affiliations:** ^1^Department of Biophysics and Human Physiology, Medical University of Warsaw, Warsaw, Poland; ^2^Department of Clinical Research, Faculty of Infectious and Tropical Diseases, London School of Hygiene & Tropical Medicine, London, United Kingdom; ^3^Complex Chronic Diseases Program, British Columbia Women's Hospital and Health Centre, Vancouver, BC, Canada; ^4^Department of Infection Biology, Faculty of Infectious and Tropical Diseases, London School of Hygiene & Tropical Medicine, London, United Kingdom; ^5^CEAUL - Centro de Estatística e Aplicações, Faculdade de Ciências, Universidade de Lisboa, Lisbon, Portugal

**Keywords:** myalgic encephalomyelitis chronic fatigue syndrome, quality control, targeted-genome association study, reproducibility, genome-wide association study

## Introduction

Myalgic Encephalomyelitis/Chronic Fatigue Syndrome (ME/CFS) is a debilitating disease characterized by persistent fatigue and post-exertion malaise, accompanied by other symptoms (1, 2). The direct cause of the disease remains elusive, but it may include genetic factors alongside environmental triggers, such as strong microbial infections and other stressors (3, 4).

With the aim to identify putative genetic factors that could explain the pathophysiological mechanisms of ME/CFS, four genome-wide association studies (GWAS) and two targeted-genome association studies (TGAS) were conducted in the past decade (5–10). In the four GWAS, thousands of genetic markers located across the whole genome were evaluated for their statistical association with ME/CFS (5–8). The two TGAS had the same statistical objective of the four GWAS, but alternatively investigated the association of the disease with numerous genetic markers located in candidate genes related to inflammation and immunity (9) and in genes encoding diverse adrenergic receptors (10). The findings from all these different studies suggested conflicting evidence of genetic association with ME/CFS: from absence of association (7), through mild association (10) up to moderate associations of a relatively small number of genetic markers (5, 6, 9). The most optimistic GWAS suggested more than 5,500 candidate gene-disease associations (8). This inconsistency in the reported findings prompted us to review the respective data. With this purpose, the present opinion paper first revisits the recommended quality control (QC) checks for GWAS and TGAS, and then summarizes which ones were performed by those studies on ME/CFS.

## Review of the Recommended QC Checks for Genetic Data

Current GWAS or TGAS of ME/CFS are based on data of the so-called single nucleotide polymorphisms (SNPs) located in specific positions of the human genome. These genetic markers are short nucleotide sequences that differ in a single position from each other. Each possible sequence of a SNP is interpreted as a different allele. In theory, there are up to four alleles of the same SNP given that there are only four possible nucleotides (A, C, G, and T). However, by design, classical genotyping technologies can only assess the two most frequent alleles per SNP. As an alternative to classical GWAS and TGAS, studies using data from next-generation sequencing technologies are able to assess all possible alleles of a given SNP. As far as we know, these alternative studies have been never performed on ME/CFS.

In general, several QC checks should be performed in the genetic data before carrying out the association analysis itself. First, it is important to determine all monomorphic SNPs and to report the respective number. These SNPs are non-informative for the subsequent genetic association analysis, because they show the same allele in all study participants. It is also important to calculate the so-called minor allele frequency (MAF) of each SNP. Statistically speaking, the MAF is defined as the frequency of the least frequent allele of a given SNP. In practice, a very low MAF is in the same order of magnitude of the underlying genotyping error rate and, therefore, SNPs under this condition should be excluded from the study. A typical threshold for a very low MAF ranges from 1 to 5%. Less stringent thresholds for the MAF can be used in studies with smaller sample sizes.

Second, the validity of the Hardy-Weinberg Equilibrium (HWE) should be tested in the observed genotype frequency distribution of each SNP. The HWE is a mathematical expectation for the probability of observing a given genotype under random mating (or panmixia), no selection, no migration, non-overlapping generations, and no genotyping errors. According to the HWE, the frequency of a given genotype is expected to be factorized into the product of the respective allele frequencies. The HWE is usually tested by the popular Pearson's χ^2^ goodness-of-fit test. In this statistical test, *p*-values below the specified significance level suggest evidence against the HWE. Since the HWE is supposed to be tested in data of each SNP separately, the significance level of each individual test should be adjusted in order to ensure a global significance level for this QC check. Bonferroni or Sidak-Dunn corrections are two popular methods to make such adjustment. Alternatively, one can use procedures based on the control of the false discovery rate, as proposed by Benjamini and Hochberg (11). In theory, deviations of the HWE can result from the genetic selection of a specific allele in patients. Because of this possibility, some researchers prefer to test the HWE using data from healthy controls alone. However, this preference has the disadvantage to decrease the power of the respective statistical test. On the other hand, a flagrant deviation of the HWE also suggests non-negligible genotype errors associated with a given SNP. Since one cannot distinguish selection from eventual genotyping errors, the SNPs with gross deviations of the HWE are typically excluded from the analysis.

Third, the proportion of heterozygous genotypes (i.e., heterozygosity rate) across all SNPs should be calculated for each individual sample. Excessive heterozygosity rate suggests a possible contamination of the respective biological sample, while reduced heterozygosity rate indicates genetic inbreeding. The usual practice is to exclude samples from individuals whose heterozygosity rates are not falling into a “confidence” band. This confidence band is usually defined by the average heterozygosity rate of all the samples plus/minus a given number of times the standard deviation of the heterozygosity rate. The heterozygosity of SNPs located in the X chromosome is also used to confirm the gender of a sample and to detect putative label swaps.

Fourth, data of SNPs or of individuals with low genotyping rates should be excluded from the analysis. The genotyping rate of a given SNP is the proportion of individuals with fully determined genotypes of that SNP, whereas the genotyping rate of a given individual is the proportion of SNPs with a fully determined genotype of that individual. A low genotyping rate of a given SNP suggests that the genomic site associated with that SNP includes another type of genetic variation (*e.g*., deletion or insertion). A low genotyping rate of a given individual indicates a low quality of the DNA material used for genotyping. Again, researchers must decide what is considered a reasonable genotyping rate for their study. In addition, different exclusion criteria can be applied to the genotyping rates of SNPs and individuals.

Additional QC checks (*e.g*., assessing the genetic distance between sampled individuals or checking their ancestry) can also be performed in GWAS and TGAS, as reviewed elsewhere (12). However, they are more relevant for large-scale population genetic studies.

## Analysis of QC Checks From Current GWAS and TGAS on ME/CFS

Table 1 summarizes the QC checks performed by each GWAS and TGAS on ME/CFS. On the one hand, the study of Perez et al. (8) only performed the QC check based on the MAF. This study also used a non-standard criterium for selecting SNPs: those with MAF <0.10 in either patients or reported in the Kaviar database were excluded from the analysis. On the other hand, Herrera et al. (7) performed all QC checks recommended for a GWAS. The remaining studies performed almost all standard QC checks with the exception of the one based on the heterozygosity rate. Interestingly, Johnston et al. (10) mentioned this QC check in the Materials & Methods of their study. However, they neither provided any specific information about how this QC was actually performed nor showed any statistical summary of the heterozygosity rate. Finally, Smith et al. (5) did not exclude any SNP based on a too-low MAF.

**Table 1:** Summary of the QC checks performed in published GWAS and TGAS on ME/CFS.

**Reference, type of study**	**Monomorphic SNPs or SNPs with low MAF**	**HWE**	**Heterozygosity**	**Genotyping rate**
Smith et al. (5), GWAS	•The total number of monomorphic SNPs was reported •SNPs were not excluded according to MAF	•The HWE was tested using data from healthy controls alone •A significance level of 0.05 was used in the statistical tests	•Heterozygosity of SNPs in the X chromosome was used for confirming gender of the samples	•SNPs with genotyping rates <80% were excluded •Individual samples with genotyping rates <92% were repeated
Schlauch et al. (6), GWAS	•The total number of SNPs with too-low MAF was reported •SNPs with MAF <0.05 were excluded	•The HWE was tested using data from both healthy controls and patients •A significance level of 0.0008 was used in the statistical tests	•Heterozygosity of SNPs in the X chromosome was only used for confirming gender	•SNPs with genotyping rates <95% were excluded •Individual samples with genotyping rates <95% were excluded
Herrera et al. (7), GWAS	•SNPs with MAF <0.01 were excluded	•The HWE was tested using data from both healthy controls and patients •A significance level of 0.00001 was used in the statistical tests	•Samples with heterozygosity rate higher or lower than two standard deviations of the average heterozygosity for all samples were excluded from the analysis •Heterozygosity of SNPs in X chromosome was also used for confirming gender	•SNPs with genotyping rates <97% were excluded. •Individual samples with genotyping rates <90% were excluded
Perez et al. (8), GWAS	•SNPs with MAF <0.10 in either patients or reported in the Kaviar database were excluded.	•Not reported	•Not reported	•Not reported
Rajeevan et al. (9), TGAS	•SNPs with MAF <0.05 were excluded	•The HWE was tested using data from both healthy controls and patients •A significance level of 0.01 was used in the statistical tests	•Not performed	•SNPs with genotyping rates <80% were excluded •Genotyping rates were performed in each individual sample
Johnston et al. (10), TGAS	•SNP with MAF <0.01 were excluded	•Not reported	•Heterozygosity was reported as a QC check but there was no information about the criterium used	•Not reported

## Discussion

This opinion paper shows partial QC checks in the majority of the published genetic association studies on ME/CFS, the exception being the study carried out by Herrera et al. (7). The assessment of the performed QC checks is essential to ascertain the quality of the respective genetic data. In this regard, the genetic data from Perez et al. (8) deserves to be further analyzed to ascertain the validity of the reported findings. Such assessment can follow the QC steps outlined here and exemplary performed by Herrera et al. (7). The remaining studies can also benefit by an additional quality check related to heterozygosity rate so that possible sample contaminations can be ruled out. The absence of this check does not immediately invalidate the genetic data of these studies. We could have done such check if the corresponding genetic data were available either in an open-access repository or as a Supplementary File within the respective publication, a data-sharing practice followed by several ME/CFS researchers (13–15). Consequently, it is unclear whether aberrant heterozygosity rates (due to sample contamination) are one of the explanations for the conflicting evidence of genetic associations reported by these studies. In this regard, Herrera et al. (7) excluded five out of their 109 samples (5%) based on the heterozygosity rate. In simple statistical applications using large sample sizes, a 5% sample contamination might be too low to have a substantial impact on the respective findings. However, in the specific context of GWAS and TGAS where stringent significance levels are used to control for multiple testing, such a level of sample contamination could reduce the underlying statistical power and leave relevant disease-gene associations undetected.

Besides the partial QC checks, the investigated genetic data on ME/CFS suffer from the curse of not having an objective biomarker for disease diagnosis. Similar problem can be envisioned for other complex diseases lacking a biomarker, such as Fibromyalgia and the Gulf War Syndrome. The absence of a biomarker is likely to introduce a possible misclassification of the true disease status of the recruited patients (16). To illustrate this putative problem, Herrera et al. (7) recruited nine obese (with body mass indexes equal or higher than 35 kg/m^2^) out of 61 patients based on the 1994 Center for Diseases Control Criteria (1) and Canadian Consensus Criteria (2). Notwithstanding controlling for the body mass index in the respective association analysis and the exclusion of known diseases, it is unclear whether the obesity observed in these patients was a direct consequence of ME/CFS or instead caused by another ongoing disease strongly associated with fatigue. A solution to this problem is to use more advanced statistical methodology where misclassification can be directly included in the data analysis (17, 18). However, given the complexity of this methodology, we argue that a stronger collaboration between the ME/CFS research community and statistical geneticists should be reached. In principle, this collaboration is expected to promote better statistical analyses, to improve data interpretations and, ultimately, a better assessment of the genetic component in ME/CFS.

In summary, given the partial QC checks performed in current GWAS and TGAS, the question of a genetic component in ME/CFS remains open for investigation. To accelerate the discovery of promising disease-gene association, future genetic studies of ME/CFS should set data and methodological standards as high as those followed by the 1,000 Human Genome Project and the UK10K project (19, 20). Data sharing should also be a general practice to provide the researcher community the opportunity to perform additional checks or alternative analyses of the same data.

## Author Contributions

NS conceptualized this research. AG and NS performed the literature review. EL and LN helped in the interpretation and discussion of all the results. All authors read, revised, and approved the final draft of the manuscript.

## Conflict of Interest

The authors declare that the research was conducted in the absence of any commercial or financial relationships that could be construed as a potential conflict of interest.

## Abbreviations

GWAS: Genome-wide association study
HWE: Hardy-Weinberg Equilibrium
MAF: minor allele frequency
ME/CFS: myalgic encephalomyelitis/chronic fatigue syndrome
QC: quality control
SNP: single nucleotide polymorphism
TGAS: targeted-genome association study.
